# CT引导下Hook-wire定位肺磨玻璃样微小结节微创切除的临床研究

**DOI:** 10.3779/j.issn.1009-3419.2014.12.04

**Published:** 2014-12-20

**Authors:** 向阳 初, 晓彬 侯, 连斌 张, 志强 薛, 志鹏 任, 佳新 温, 毅 刘, 克峰 马, 玉鹗 孙

**Affiliations:** 100853 北京，解放军总医院胸外科 Department of Thoracic Surgery, PLA General Hospital, Beijing 100853, China

**Keywords:** 肺磨玻璃样结节, CT引导下Hook -wire定位, 微创切除, Ground glass opacity, Computed tomography-guided localization with a hook-wire system, Minimally invasive resection

## Abstract

**背景与目的:**

肺磨玻璃样微小结节（ground glass opacity, GGO）病灶的定位是微创手术切除的技术难点。各种定位方法均有报道，但每一种方法均有其不足。本研究拟通过评价术中CT引导下Hook-wire定位对GGO微创切除的价值，初步探索肺部 < 10 mm的GGO积极手术治疗的必要性和可行性。

**方法:**

2009年10月-2013年10月共32例GGO患者，41个GGO，行胸腔镜微创切除术，麻醉插管后皆在手术体位下行计算机断层扫描（computed tomography, CT）CT引导Hook-wire定位。记录术中CT引导下Hook-wire定位技术的失败率、并发症、胸腔镜手术转为开胸手术的几率、住院时间等，计算病灶组织学分型中的恶性几率，讨论肺部 < 10 mm的GGO积极手术治疗的必要性。

**结果:**

共32例患者（男性15例，女性17例）行41个GGO胸腔镜微创切除术，其中2个病灶、3个病灶和5个病灶同时微创切除患者数量分别是3例、1例、1例。病灶直径2 mm-10 mm（平均5 mm），病灶距离胸膜垂直距离5 mm-24 mm（平均12.5 mm）。术中CT引导下Hook-wire定位成功率100%，严重并发症发生率0，转化为开胸手术比率为0，CT定位时间平均8.4 min（4 min-18 min），微创切除病灶所需时间平均32 min（14 min-98 min），中位住院时间为8 d（5 d-14 d）。GGOs术后组织学诊断结果为：原位腺癌（肺泡癌）19例，约46.3%，腺癌8例，约19.5%，大细胞癌1例，约2.4%，不典型腺瘤样增生9例，约22%，炎性病灶4例，约9.8%。

**结论:**

肺部GGO是恶性病灶的几率很大，对典型GGO患者积极微创手术治疗是非常必要的；术中CT引导下Hook-wire定位技术极大提高GGO微创切除可行性、并发症发生率低，对于GGO的鉴别诊断及治疗具有很好的临床价值。

## Abstract (en)

**Background and objective:**

Localization of pulmonary ground glass small nodule is the technical difficulty of minimally invasive operation resection. The aim of this study is to evaluate the value of intraoperative computed tomography (CT)-guided localization using a hook-wire system for small ground glass opacity (GGO) in minimally invasive resection, as well as to discuss the necessity and feasibility of surgical resection of small GGOs (< 10 mm) through a minimally invasive approach.

**Methods:**

The records of 32 patients with 41 small GGOs who underwent intraoperative CT-guided double-thorn hook wire localization prior to video-assisted thoracoscopic wedge resection from October 2009 to October 2013 were retrospectively reviewed. All patients received video-assisted thoracoscopic surgery (VATS) within 10 min after wire localization. The efficacy of intraoperative localization was evaluated in terms of procedure time, VATS success rate, and associated complications of localization.

**Results:**

A total of 32 patients (15 males and 17 females) underwent 41 VATS resections, with 2 simultaneous nodule resections performed in 3 patients, 3 lesion resections in 1 patient, and 5 lesions in a patient. Nodule diameters ranged from 2 mm-10 mm (mean: 5 mm). The distance of lung lesions from the nearest pleural surfaces ranged within 5 mm-24 mm (mean: 12.5 mm). All resections of lesions guided by the inserted hook wires were successfully performed by VATS (100% success rate). The mean procedure time for the CT-guided hook wire localization was 8.4 min (range: 4 min-18 min). The mean procedure time for VATS was 32 min (range: 14 min-98 min). The median hospital time was 8 d (range: 5 d-14 d). Results of pathological examination revealed 28 primary lung cancers, 9 atypical adenomatous hyperplasia, and 4 nonspecific chronic inflammations. No major complication related to the intraoperative hook wire localization and VATS was noted.

**Conclusion:**

Intraoperative CT-guided hook wire localization is useful, particularly in small GGO localization in VATS wedge resection and has a significantly low rate of minor complications. Lung GGOs carry a 90% risk of malignancy. Aggressive surgical resection of these GGOs is necessary and feasible through the guidance of intraoperative CT localization technique.

肺内磨玻璃密度影（ground-glass opacity, GGO）是指高分辨率计算机断层扫描（high-resolution computed tomography, HRCT）图像上表现为密度轻度增加，但其内的支气管血管纹理仍可显示。此类病灶多数没有明确的临床症状。目前国内外学者^[1, 2]^发现此征象常为肺部疾患的早期表现，特别是肺部腺癌的早期表现。国内外许多研究^[3, 4]^显示，GGO具有较高的恶性率，多数是原位腺癌，亦可是微浸润腺癌，甚至是浸润腺癌。临床实践中，CT引导穿刺活检和电视辅助胸腔镜手术（video-assisted thoracic surgery, VATS）微创手术切除是获得组织标本的常用手段。但是，VATS手术对于直径 < 10 mm、距离胸膜 > 5 mm的GGO有63%的活检失败率^[5]^。尽管如此，对于10 mm以下的小GGO，VATS手术可能是唯一可行的方法，而其中的技术难点就集中在GGO病灶的定位问题。对于GGO的微创切除定位技术的研究有很多^[6-11]^。但是各种方法都有其局限性。CT引导穿刺针定位最大的问题是在患者转运和术中单肺通气过程中的定位针脱落问题。我们报道的术中CT引导Hook-wire穿刺定位技术很好地解决了这一问题，以下为该研究的初步报告。

## 1 材料和方法

术中CT装置（Simens Somatom Sensation Open with Sliding Gantry, Simens Medical System, Germany）见图 1。全麻双腔气管插管成功后取手术体位，自制定位器置于目标病灶大概位置的胸壁体表（图 2），行CT扫描，确定病灶所在横断面和定位器交叉点作为穿刺点，结合肋骨与病灶之间的关系确定穿刺角度，尽量选取垂直于体表的方向穿刺定位，测量所需穿入的深度，穿刺针套管长10.7 cm，针芯长20 cm（DuaLok^®^, Bard Inc., United Kingdom）（图 3）。穿刺完成后立即行CT扫描明确穿刺针和病灶之间的关系（图 4），位置合适后释放套管，剪去外露部分穿刺针，VATS手术常规采用我科首创的单操作孔手术方式（图 5），并使用强生或美外公司生产的切割缝合器楔形切除病灶。标本切除后立即切开肺组织明确病灶已被成功切除（图 6），送冰冻病理，如果确定是非小细胞肺癌，则立即行肺叶切除并纵隔淋巴结清扫术；若为良性病灶则常规关胸。VATS手术时间定义为切皮开始到明确切下的肺组织中包含目标病灶。将患者信息、GGOs的病理结果及定位成功率、VATS转为开胸手术比率、VATS持续时间及术后并发症进行分析。

**1:**
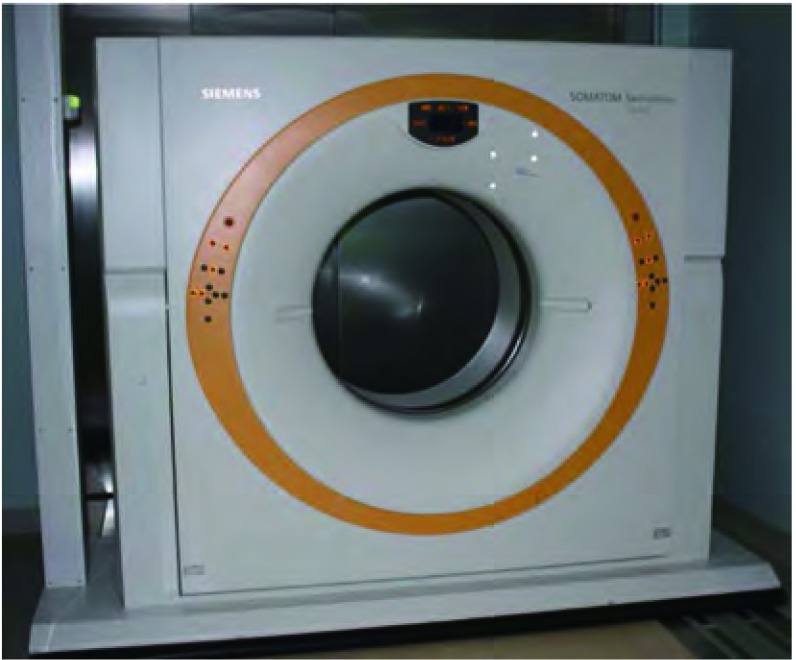
手术室中电子计算机断层扫描（CT）设备 Computed tomography (CT) scanner

**2:**
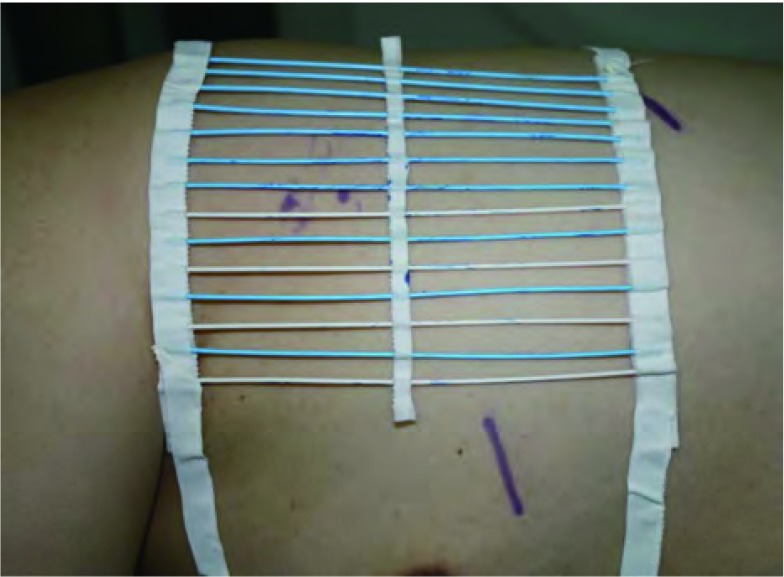
自制定位器 Homemade locator

**3:**
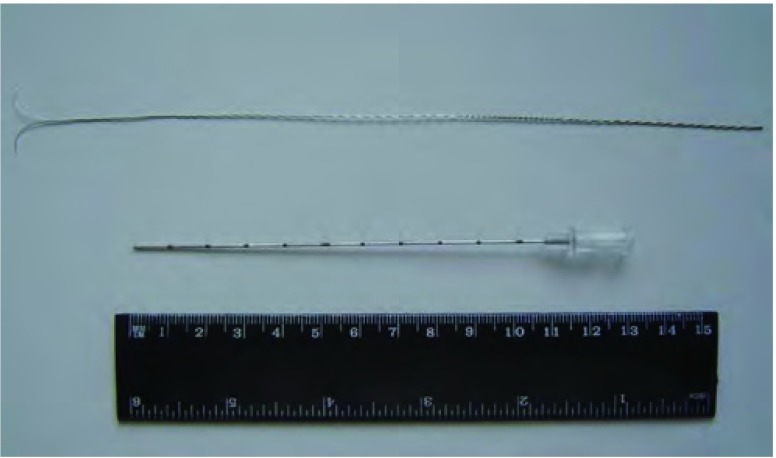
Hook-wire定位针 Hook-wire pilot pin

**4:**
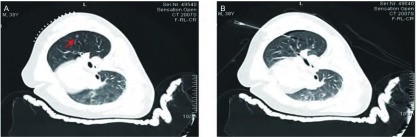
穿刺针定位于肺磨玻璃样微小结节（GGO）病灶的CT扫描图。A：自制定位器和GGO病灶；B：穿刺针定位于病灶。 CT scanograms of the ground glass opacity (GGO) lesions with pilot pin. A: Homemade locator and GGO lesions; B: Lesions with pilot pin.

**5:**
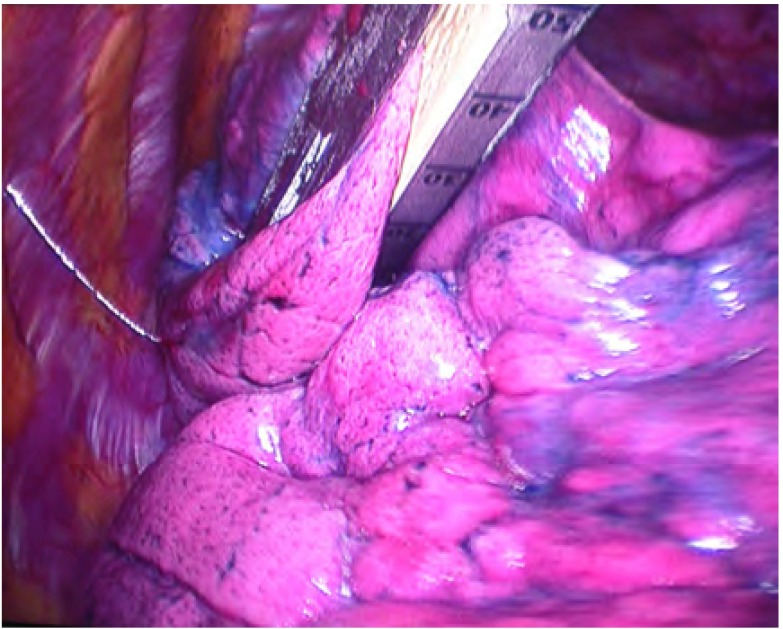
Hook wire定位针引导肺部GGO病灶的电视胸腔镜手术（VATS）切除 The video-assistant thorascope (VATS) resection of GGO lesions guided by Hook-wire pilot pin

**6:**
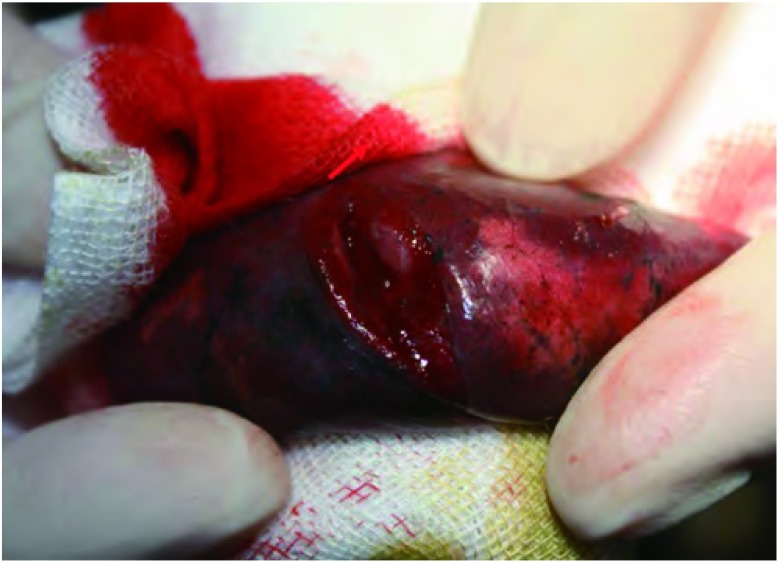
切除的肺组织中见直径4 mm病灶 Lesions (d=4 mm)

## 2 结果

2009年10月-2013年10月共32例患者，41个微小结节，行胸腔镜切除术，其中3例患者同一肺叶2个结节，1例患者同侧肺3个结节，1例患者同侧肺5个结节，男性15例，女性17例，平均年龄54岁（24岁-67岁），结节位于左肺12例（29.3%），位于右肺20例（70.7%）。

CT引导Hook-wire定位时间为4 min-18 min，平均耗时8.4 min。41个病灶中只有5个（12.2%）是Hook-wire定位针直接准确穿刺到病灶中，其余36个（87.8%）病灶均是穿刺到病灶周围，但均在10 mm范围之内，且对手术切除无任何影响。Hook-wire定位针定位成功后无任何延误即行VATS手术，VATS手术时间平均32 min（14 min-98 min），所有GGO病灶均成功切除（100%），无中转开胸病例。

术中最常见的并发症是CT扫描发现的极少量气胸（11例，34.4%）和肺内出血（5例，15.6%），但因为VATS手术即刻进行，对患者及手术过程无任何不利影响。

41个GGO病灶病理类型见表 1。

**1:** GGOs病理类型分析 Pathological analysis of GGOs

Pathological analysis	GGO (*n*)	Percent (%)
Primary lung cancer	28	68.3
AAH	9	22
Non-specific chronic inflammatory lesions	4	9.7
AAH: atypical adenomatoid hyperplasia.		

## 3 讨论

随着高分辨率CT的广泛普及，肺部GGO的检出率逐年增加。对于肺部微小结节的病理活检，VATS手术是广泛采用的手术方式。但是，对于距离胸膜比较深、亚厘米的微小结节以及一些低密度的GGO，由于VATS术中难以准确定位，从而导致比较高的开胸手术比率。对于此类病灶，术前首先进行病灶的定位非常必要。Nakashima^[12]^提出病灶定位的标准包括：①结节最大径≤5 mm；②结节最大径与结节到脏层胸膜之间最小距离之比≤0.5；③化疗后CT扫描仍可见低密度结节影。他建议若肺部结节符合以上两个以上标准就应在VATS术前定位。各种定位方法均有报道^[13-16]^。每一种方法均有其不足：在结节周围肺实质内注射甲基蓝可能会导致胸膜以及胸腔内染料着色的风险，从而使得后面进行VATS的术者很难辨认具体的病灶位置；而就超声定位而言，一是超声分辨率比较低，难以很好观察和定位亚厘米的结节特别是GGO结节，二是术中超声对于操作者的依赖性比较高，需要具有丰富经验的操作者并且需要被检查的肺完全塌陷才能很好地定位，而肺完全塌陷的患者术后恢复不良，另外肺气肿患者的肺也很难使其完全塌陷。

我们术中CT引导Hook-wire穿刺定位的前期研究取得了令人鼓舞的结果。Chen等^[17]^也曾报道此种定位方法，但由于我们在手术室取手术体位进行穿刺定位，避免了患者的转运和搬动，定位针脱落问题得到很好解决；另外，由于VATS手术在定位结束后即刻进行，穿刺可能导致的气胸乃至血胸造成严重后果的风险几乎可以降低到0。本组病例CT定位成功率为100%，所有GGO得到明确的病理诊断。

有报道^[18]^术前病灶定位使VATS手术时间由180 min明显降低至90 min。我们本组病例在病灶定位后VATS手术平均耗时32 min，明显低于该报道。

本研究中，所有病例未出现严重并发症。只有1例定位针脱落，但因为是手术中直视下扯脱，且肺表面针眼清晰可见，对手术切除未产生任何影响。比文献^[19, 20]^报道的穿刺针脱落几率明显减低，有少部分患者在穿刺后出现少量气胸和肺内出血的情况，但因为我们VATS手术即刻进行，处理此类轻微并发症无任何难度，由此可能造成的严重后果风险为0。

GGO组织学类型中约90%为原发性肺癌或癌前病变，这与多数报道结果一致。因此，积极的手术处理对于此类病例非常必要性。虽然我们的研究病例数尚少，但对于小GGO的微创切除提供了技术上的可行性，值得进一步推广。
